# Quantification of Porous Properties of Shear Crystallized Lipids

**DOI:** 10.3390/molecules27030631

**Published:** 2022-01-19

**Authors:** Brandon D. Howard, Farnaz Maleky

**Affiliations:** Department of Food Science and Technology, The Ohio State University, 2015 Fyffe Court, Columbus, OH 43210, USA; howard.1448@osu.edu

**Keywords:** crystallized lipids, shear, cocoa butter, trilaurin, monostearate, pore network, connectivity, pore size

## Abstract

The aim of this study was to investigate the impact that shear and composition have on the structural properties associated with the porous phases of lipids. To accomplish this aim, we developed eight main crystallized samples using cocoa butter (CB) or trilaurin (TL) in the presence or absence of monostearate (M) (5% *w*/*w*). The samples were sheared at 500 s^−1^ using random (RS) or laminar (LS) shear at a cooling rate of 2 °C/min. Using the maximal ball (MB) algorithm, several empirical void measurements such as connectivity (z), pore and throat volume weighted radii (*R*_43_), and void fraction (v) were quantified using 3D X-ray microcomputed tomography images. Most void features were identified as micropores (R ≥ 10 μm) possibly originating from the crystallization process and post-process crystal growth. Likewise, depending on the applied treatments, mechanisms impacting void formation were found to produce noticeable variation in v (0.019 to 0.139) and to determine whether void morphology was spherical, irregular, and/or highly connected.

## 1. Introduction

The nature of crystallized lipids is highly dependent on the treatments applied to them during and after crystallization. For example, by adjusting the shearing and cooling rates, or altering the composition, it is possible to engineer crystallized lipids with a variety of structural and thermodynamic properties [1,2]. The manipulation of these parameters often affects the crystal aggregation and growth kinetics of lipids during crystallization, which may result in changes to crystal size and shape, polymorphic behavior, phase ratio, and chemical nature [3,4,5,6,7,8,9,10]. Specifically, it affects their kinetic and thermodynamic barrier properties due to differences in orientation and spacing between crystals, the tortuousness of the path taken by diffusing penetrants, the number of phases available for migration, and the solubility of the network [11,12,13,14,15].

Typically, high shearing rates can enhance the barrier properties of lipids due to the rupture of large crystals and the formation of a more compact crystal network [7,14,16,17,18,19]. Moreover, differences in network properties can be obtained depending on the shear applied. Previously, it has been found that the application of laminar shear can orient crystals parallel to one another during crystallization [7,20,21]. Maleky and Marangoni (2011) applied this kind of shear to cocoa butter and observed a rapid crystallization that produced crystalline networks with higher constrictivity and tortuosity compared to those processed under static and random shear [12]. The enhancement of these structural features inevitably helped to lower the diffusion and permeability coefficients of oil through the network, making the application of certain shears an ideal choice for engineering the structural properties of lipids for the minimization of mass transfer.

However, when one considers the migration of moisture through lipids, mechanisms attributing to mass transfer are not confined to the crystalline and oil phase alone. Studies that have imaged the surface of lipids or utilized mercury porosimetry have suggested the presence of significant internal porous structures at several length scales [22,23,24,25]. This finding was confirmed when Paluri et al. (2017) nondestructively imaged the internal porous structures of lipids using X-ray computed tomography [15]. This imaging technique enabled accurate measurements of the void fraction and the development of a new model that helped assess effective diffusion coefficients through the void and oil phases [26]. The use of this model on trilaurin and triolein blends found that the void phase played a predominant role on the diffusion of moisture in networks with as high as 40% (*w*/*w*) added liquid oil and as low as 1% void fraction. These results show the significant influence of void fraction on moisture migration and reiterate the importance of identifying morphological features critical to the barrier properties of the void, which impact the functionality of lipids. Moreover, the quantity and morphology of the void are expected to affect the rheology and/or thermal properties of lipids [27,28]. Thus, to assess the impact of the void on the properties and performance of lipid-based materials, we must first understand the impact of processing and composition on the inclusion and morphology of voids.

This study assesses the impact of random and laminar shear and lipid composition on the development of porous lipid networks. To accomplish this goal, two different fats—trilaurin (a monoacid triacylglycerol, TAG) and cocoa butter (a mixed acid TAG)—were crystallized at a constant cooling rate using two different types of shear attachments in the presence or absence of monostearate. Emulsifiers were added to the sample because they can aid in the development of air/oil foams and alter the crystallization kinetics and structure of lipids [29,30]. Crystallized samples were then analyzed for their polymorphic, thermal, and pore network properties.

## 2. Results and Discussion

**Figure 1:**
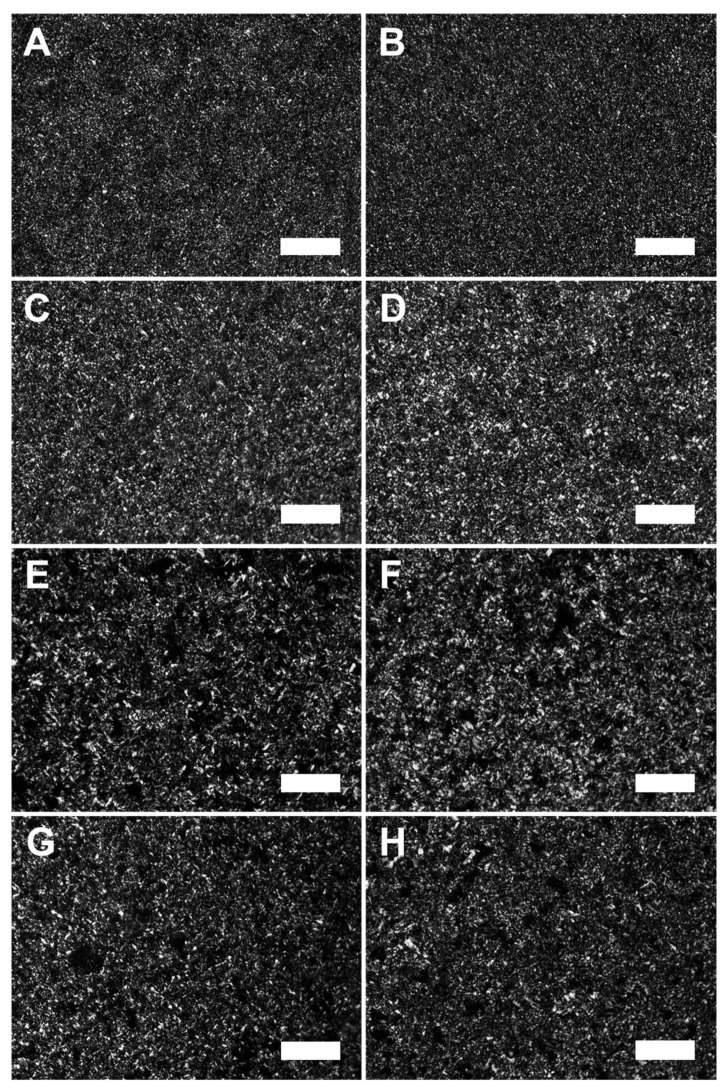
Representative PLM images for cocoa butter (**A**–**D**) and trilaurin (**E**–**H**) samples imaged at 10× magnification. (**A**) represents CB RS, (**B**) CB LS, (**C**) CB/M RS, (**D**) CB/M LS, (**E**) TL RS, (**F**) TL LS, (**G**) TL/M RS, and (**H**) TL/M LS. The white scale bar at the bottom right corner of each image represents 100 μm in length.

Images of crystal microstructure obtained by PLM for lipid samples processed under 500 s^−1^ are reported in Figure 1A–H. The images clearly show that, regardless of the composition or the type of shear, the treatments created compact crystal networks with small, finely dispersed crystals. To determine whether there were differences in crystal size, quantitative measurements of PLM images and the samples’ SFC were performed and reported in Table 1. This analysis revealed that CB samples processed under RS and LS had crystal sizes of about 8 to 8.5 μm^2^ and those with the addition of monostearate had crystal sizes of 11.4 to 13.3 μm^2^. The increase in crystal size for commercial fats such as CB with the addition of an emulsifier has been previously observed with the addition of lecithin to CB and palm oil [14,31]. This increase is generally thought to occur because emulsifiers form thermodynamically favorable micellular structures that can lower the energy barrier for nucleation [32]. The reduction in the energy barrier in combination with the increase in the number of surfaces made available for such events can lead to an increase in the rate of secondary nucleation and, therefore, an increase in crystal size [1]. For mono fatty acid triacylglycerol networks such as TL, the addition of monostearate and changes in the applied shear had little to no effect on crystal sizes with measurements ranging between 13.6 and 18.6 μm^2^. This result agrees with previous findings showing that, when added to TL, monoacylglycerols with a high carbon content will cause little to no increase in crystal growth rates [33]. Likewise, the similarity of crystal size in RS and LS samples of each fat may suggest that microscale crystal growth is more affected by the composition and shearing rate rather than the shear type.

**Figure 2:**
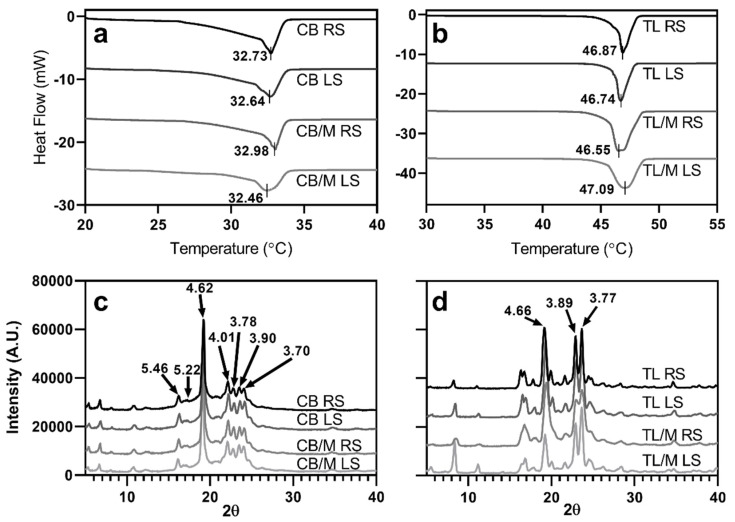
Plotted DSC (**a**,**b**) and XRD (**c**,**d**) data for cocoa butter and trilaurin samples. Different colored lines represent the melting profiles and diffractograms of the lipid blends. Straight lines passing through thermogram peaks with corresponding temperatures represent the melting point temperatures for respective samples. Values with corresponding arrows pointing to peaks in sample diffractograms represent d-spacings between planes of symmetry measured in angstroms (Å). Plots for DSC and XRD were created by plotting replicates that were most representative of the average observed melting profile and diffractogram, respectively.

To further elucidate how the composition and shear impact fat crystal properties, the analyzed thermal and X-ray diffraction data are reported in Figure 2. As seen, differences in fat composition and shear produced thermodynamically stable networks with melting point temperatures of about 32.5–33 °C for CB and 46.5–47 °C for TL. Likewise, X-ray diffraction results showed CB had six main peaks varying in sizes with d-spacings between 3.70 and 5.46 Å, whereas TL diffractograms had three large peaks ranging between 3.77 and 4.66 Å. Crystallized CB and TL with corresponding melting points and d-spacings reported here indicate the presence of the β_v_ and β polymorph, respectively [34,35,36]. These results suggest that regardless of the additives and shear type applied, the processing of both CB and TL created highly stable crystalline networks for all samples. It also corroborates previous findings that the addition of shear and emulsifiers can accelerate the transition of lipids to stable polymorphs [5,29,37]. Moreover, PLM (Figure 1A–H) and polymorphic data (Figure 2a–d) confirmed that all networks were highly compact with homogeneously dispersed crystals (<20 μm^2^) comprised of stable polymorphs. However, the morphology of crystals and their subsequent aggregation behavior (shown as crystal size and SFC data in Table 1) is dependent on the chemical nature of lipids and whether external shear and temperature gradients are applied [7,38,39,40]. This dependency suggests that variations in composition and applied shear can significantly impact network forming mechanisms, the extent of their influence, and the length scales in which they are associated. This impact is particularly true for pore network properties that are directly impacted by crystallization dynamics at submicron to macroscopic length scales [24,25]. Since void structures are strongly correlated with structural properties of lipids at all length scales, it was expected that detailed measurements of the void would allow further delineation of their influence. Thus, to further elucidate these influences, the remainder of this discussion focuses on measurements of the voids obtained from CT images.

**Figure 3:**
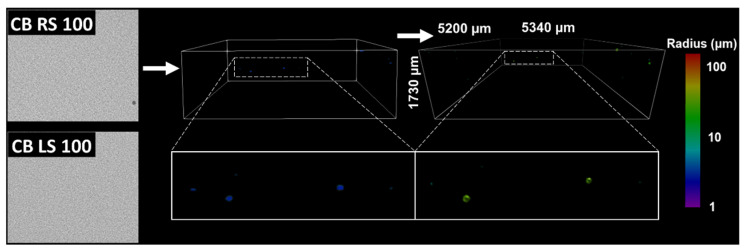
Pore network visualizations for 100 s^−1^ cocoa butter samples. (**Left**) CT images slices, (Middle) raw 3D plots (blue) of CB RS 100 void and (**Right**) a 3D ball and stick plot of CB RS 100 pores and throats after MB analysis. No 3D visualizations for CB LS 100 were created because these networks have no voids.

As mentioned in the crystallization methods, cocoa butter was initially crystallized under 100 s^−1^ shear to observe the impact of low shearing rates on void formation, and whether significant voids could be incorporated at these conditions. Grayscale image slices obtained by CT analysis and corresponding 3D images of raw voxels and their subsequent maximal ball networks are depicted in Figure 3 for CB samples processed with 100 s^−1^ under random and laminar shearing. In this figure, the blue 3D images are representations of the original or raw voxels of a given replicate. These images act as a standard for comparing against MB images to confirm the accuracy of the method. As is mentioned in the CT methods, the MB algorithm discretizes the pore space into maximally inscribed spheres along the medial axis of the void. Pores and throats were identified by local maximums and minimums in the maximal ball distance map (maximal ball radii), respectively [41]. Data obtained from this analysis in the form of spheres and cylinders were then used to represent the pore space in the MB images reported in Figure 3. The MB visualizations were colored using a log_10_ scale corresponding with the magnitude of radius measurements for individual pores and throats.

**Figure 4:**
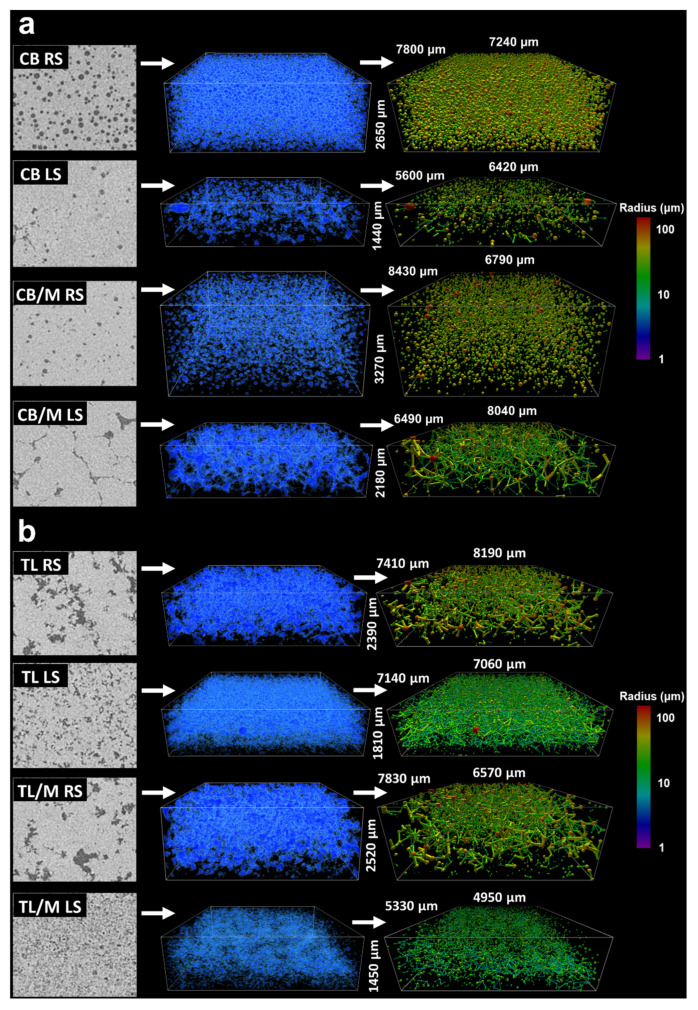
Pore network visualizations for 500 s^−1^ sheared samples, (**a**) cocoa butter and (**b**) trilaurin. Images corresponding to each sample are depicted as (Left) CT image slices, (Middle) raw 3D plots (blue) of void voxels prior to analysis, and (Right) a 3D ball and stick plot of pores and throats after MB analysis. Pores and throats are colored according to radius measurements on a log_10_ scale.

For CB samples processed under 100 s^−1^, it is obvious that little voids were incorporated during crystallization (Figure 3). Samples produced under RS had observable but extremely low quantities of heterogeneously distributed voids. LS samples had no voids present at the resolutions imaged and could not be represented by 3D images. This result suggested that RS more favorably incorporated voids than LS, likely due to it contributing axial shear that could whip air into the melt throughout crystallization. However, the lack of voids in samples developed at this lower shearing rate made it difficult to investigate in detail the impact of processing conditions on their morphology. Utilizing high shearing rates has been generally shown to increase the void fraction of foams, butters, and ceramics [42,43,44]. Likewise, these conditions may better mimic industrial processing applications that typically crystallize lipids under high shear [45]. Therefore, the CT images of the samples crystallized at a higher shearing rate of 500 s^−1^ were used for void analysis. CT images and visualizations of these CB and TL samples are shown in Figure 4a,b, respectively.

**Figure 5:**
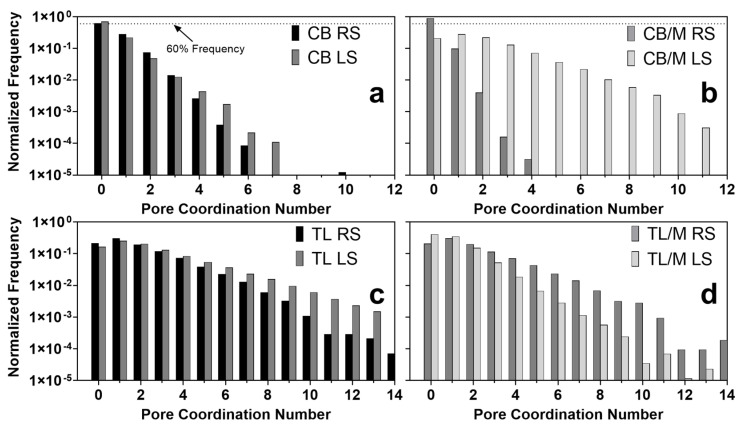
Pore coordination number distributions for 500 s^−1^ sheared samples, (**a**,**b**) cocoa butter and (**c**,**d**) trilaurin. Distributions presented were computed by taking an average of replicate distributions to represent the average distribution observed.

**Figure 6:**
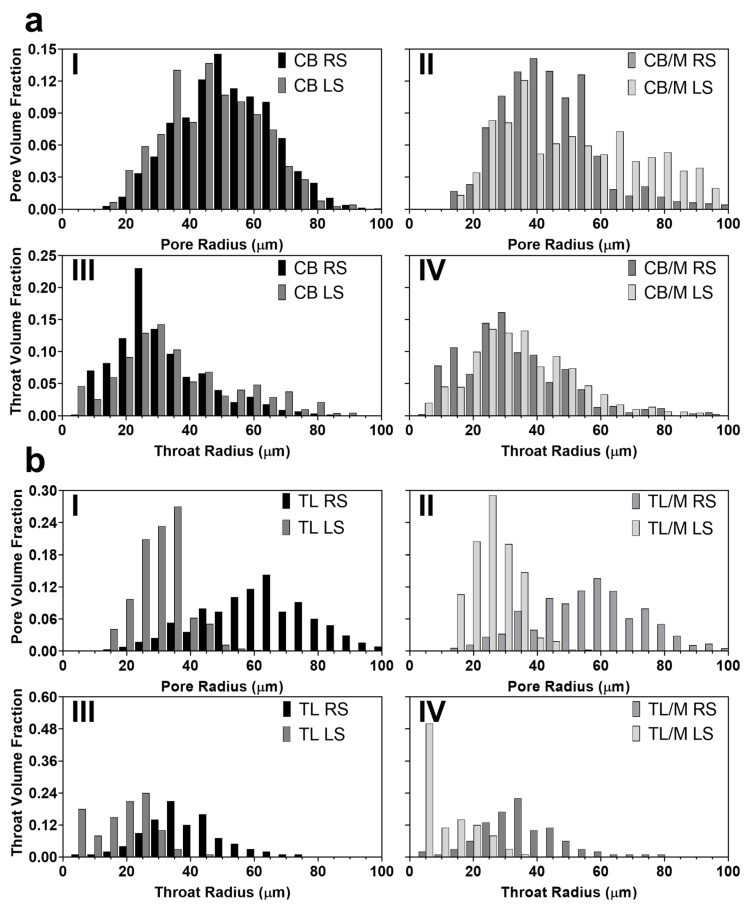
Volume weighted pore (R_43,p_) and throat (R_43,t_) radius distributions for random and laminar sheared samples: (**a**) cocoa butter and (**b**) trilaurin in the presence (**II**,**IV**) and absence (**I**,**III**) of monostearate. Bins used for each radius distribution include values within a 5 μm range centered at their nearest respective tick marks. Distributions presented were computed by taking an average of replicate distributions to represent the average distribution observed.

To elucidate mechanisms that contribute to the observed differences in the morphology of the higher-sheared samples, pore coordination numbers and pore and throat radii were quantitatively measured using the MB algorithm for each void feature. Pore coordination numbers were defined as the number of throats or paths stemming from a single pore [41]. These values along with the corresponding average coordination numbers or connectivity (z) of the void within a porous medium provide a means for describing the overall topology of porous materials and elucidating mechanisms leading to final network properties and functionality [46,47]. For radius measurements, it has been previously found that weighting corresponding distributions by the total occupied volume for void features rather than frequency, improves predictions of transport properties through porous media [48]. Likewise, porous structures that take up more volume may have a greater impact on overall material properties of lipids. Therefore, to gain additional insight into void features with sizes that most likely affect the final network characteristics and functionality, radius distributions were weighted accordingly. Pore coordination number distributions for all samples are shown in Figure 5, and pore and throat volume weighted radii distributions are presented in Figure 6. The average values for each of these distributions along with their void fraction were computed and are reported in Table 2.

Image slices and 3D visualizations of 500 s^−1^ CB samples showed spherical pores for both CB RS and CB LS (Figure 4a). However, the void of RS samples was more homogeneously distributed with about six times higher void fraction than those processed under LS (Table 2). The higher incorporation of spherical pores into RS networks is similar to what was observed for 100 s^−1^ CB samples, where axial shear induced by the impeller geometry helped incorporate a higher quantity of air bubbles during crystallization. These results confirmed that both shearing rate and type can increase the void fraction of the final crystallized network, as previously predicted. Additionally, pore and throat radii distributions in Figure 6aI,aIII revealed that CB RS and CB LS void features were similar in size and that their average pore radius (50.0 to 50.8 μm) and throat radius (34.4 to 44.4 μm) are not significantly different (Table 2). Likewise, coordination number distributions (Figure 5a) and corresponding connectivity values (Table 2) ranging between 0.39 and 0.42 for CB RS and CB LS revealed that a majority of pores were not connected to any other pore. The similarity in void features observed at this higher shearing rate may negate the effects of shear type on CB pore morphology. This finding supports what was observed in 3D visualizations, where most void features were air bubbles inserted during crystallization. Notice that CB RS and CB LS coordination number distributions followed a sharp linearly decreasing log_10_ distribution in which pores with high coordination numbers existed at low frequencies.

Void structures may alternatively develop from mechanisms driving the formation and displacement of the solid boundary during crystallization, such as sedimentation [49], liquid-phase sintering [50], and even cracking [51]. Therefore, the presence of highly connected pores may suggest that alternative mechanisms may also be contributing to the extent and development of final structural characteristics of the void other than the incorporation of air bubbles. Interestingly, the addition of monostearate to CB significantly changed the pore network of both CB RS and CB LS samples (Figure 4a). When monostearate was added to CB RS, a similar well-dispersed spherical void geometry with a significantly lower void fraction was observed. For CB/M RS the average void fraction value decreased by approximately 80%, with corresponding values around 0.022.

In contrast, samples in Figure 4a showed a strikingly different morphology when monostearate was added to CB crystallized under LS. As seen, the void of CB/M LS image slices had crack-like projections that extended throughout the 3D structure to form clearly connected voids. Pore coordination distributions (Figure 5b) and connectivity values (Table 2) showed that more than 80% of CB/M LS pores were connected to at least one other pore with a connectivity of 1.88. This result is in contrast with the spherical geometry observed for CB RS, CB LS, and CB/M RS and their respective coordination number distributions (Figure 5a,b) that showed about 60% or more of their pores not connected to any other pore. Interestingly, despite the different morphology between CB/M RS and CB/M LS, pore and throat radii distributions (Figure 6aII,aIV) showed similar measurements between void features. In fact, average values reported in Table 2 revealed that pore and throat sizes among all CB samples were similar, with values ranging between 40.2 to 50.8 and 28.8 to 44.4 μm, respectively.

To further investigate the extent to which shear and composition affect the void forming mechanisms and, in turn, the structural properties of lipids, crystallized samples of TL with and without monostearate (previously described) were also analyzed using CT. Due to the unique polymorphic and crystallization behavior of pure fats (particularly monoacids TAGs) [1], trilaurin was crystallized under the same high shear to produce a porous network possibly dissimilar to that found in cocoa butter. As shown in Figure 4, the voids of TL samples with no monostearate more nearly resembled those of CB/M LS samples. They had highly connected voids with large void fractions and observable differences in void geometry and size (depending on shearing type). The high level of connectivity observed in visualizations was in accordance with coordination number distributions (Figure 5c), which illustrate the connection of a majority of pores to at least one other pore. Connectivity values reported for TL RS and TL LS (Table 2) ranged between 1.87 and 2.44. Moreover, void fraction measurements showed that both TL RS and TL LS had similarly large void fractions between 0.118 to 0.139. This result disagreed with trends observed for void fractions of CB RS and CB LS and may suggest that, unlike cocoa butter, trilaurin crystallized at high shearing rates will develop a large quantity of voids regardless of the type of shear applied.

Regarding void morphology, CT image slices for TL RS samples (Figure 4b) revealed the development of large void structures with an irregular fractal-like cross-sectional geometry. TL LS samples showed a similar appearance with smaller void cross-sections and greater variation in geometry (from irregular to particle-like). These relative differences in the size of void features were confirmed by pore and throat radius distributions (Figure 6bI,bIII), which showed stark differences in their sizes. The average pore and throat radii reported in Table 2 were around 61.2 and 33.8 μm for TL RS and 31.8 and 18.0 μm for TL LS. Unlike CB, these changes in the size of trilaurin’s void features from different shear attachments indicate the significant role of shear type in the sample’s void development. Previously, Lencki and Craven (2012) developed porous TL networks using a wide range of supercooling temperatures and used density measurement for void analysis. They found TL crystallized under low degrees of supercooling produced networks with the highest void fractions or lowest densities [24]. They primarily attributed their finding to the production of negative pressure zones caused by the displacement of oil as triacylglycerols crystallize to the surfaces of surrounding crystals. The vacuum created from the migration of liquid oil in addition to it condensing to a crystalline form in tandem causes the formation of the void phase. This observation may explain why fats containing significantly higher SFCs or higher quantities of high-melting-point triacylglycerols will generally have higher quantities of void [15]. Rather than the surrounding spaces between crystal aggregates being occupied primarily with oil (like with CB), fats containing greater concentrations of high-melting-point triacylglycerols (such as TL) are likely to crystallize within the surrounding matrix and develop voids.

In this study, this difference can be further explained by the highly connected void morphology found in TL samples that corresponds with a highly connected or sintered crystal network. Sintering may occur from the formation of solid crystalline bridges between aggregates of similar polymorphs or the flocking together of crystals mediated by adhesion forces [40]. The formation of sintered aggregates at supersaturated crystallization conditions, such as those implemented here, occurs due to the liquid portion being more soluble than the existing solid phases [50]. This solubility in turn would be expected to cause liquid oil to crystallize at surfaces of the existing crystals, which causes swelling of the crystal phase and the formation of voids as previously mentioned. This method of void formation could make sense for TL because pure fats are known to require high supersaturations for primary nucleation to occur [1]. Thus, under the moderate supersaturations used here for crystallizing TL, one may expect that once primary nucleation began, the previously formed crystals in the melt act as catalyzing surfaces, and favor crystal growth over primary nucleation. In consequence, due to its high melting properties, TL develops high-SFC sintered crystalline networks in conjunction with highly connected voids. Since the void developed under this mechanism created long continuous structures throughout TL samples, it seems reasonable to expect that this mechanism may only predominate when significant crystallization occurs during incubation (after shearing has ended) to allow the formation of the gel scaffolding upon which crystallization may continue. Consequently, this finding may imply that the differences in the type of shear used altered crystallization behavior during incubation and were, therefore, the reason for observed differences in the size of void features.

Studies have already shown the disordered arrangements of lipid crystals developed under RS and the well-defined alignments of crystals in samples crystallized under LS [6,7]. Due to the disorder cultivated within the structural microelements of lipids processed by RS, it has been shown that slower rates of crystallization during incubation may follow [6]. The innate differences in crystal arrangement promoted by these different shears can also significantly reduce the rate of mass transfer through crystalline networks as seen in CB [12]. This reduction may justify the observed differences in the sizes of void features developed in response to sintering mechanisms. Voids developed under sintering mechanisms are dependent not only on the displacement of liquid oil but also its crystallization to surrounding crystal surfaces. Consequently, to achieve the large, microscopic differences in the size of void features observed between TL RS and TL LS (~30 μm difference in pore radius), differences in the displacement and/or oil diffusion during incubation must have occurred. With slower rates of crystallization and/or higher rates of mass transfer potentially prevailing in TL RS networks, it is likely that, in accordance with sintering mechanisms, a higher degree of oil displacement resulted prior to crystallization and created the larger void features.

The development of connected voids was also observed in trilaurin samples containing monostearate (Figure 4b), regardless of their processing conditions. TL/M RS samples created void structures with a nearly identical topology and quantity to corresponding samples without emulsifier. This finding was indicated by their similar fractal-like cross-sectional geometry observed in CT image slices (Figure 4b), in addition to their similar connectivity, pore and throat sizes, and void fractions (Table 2). The addition of monostearate to TL LS resulted in a reduction of void fraction by 37% and connectivity by 57%. Pore and throat sizes for TL/M LS were similar to those for TL LS, which also meant that all TL samples processed under RS had significantly larger void features than those processed with LS. Likewise, when comparing the SFC data of TL containing monostearate (Table 1), both TL/M RS and TL/M LS showed a 4% and 2.5% reduction in SFC from 98.2% and 97.8%, respectively. This reduction in SFC is likely the result of molecular incompatibility between monostearate and TL triacylglycerols creating irregularities within the crystal lattice [52]. Reductions in SFC in TL networks have been previously linked to changes in void fraction by Paluri et al. (2017), who found that by adding triolein (oil) to TL in as much as 40% (*w*/*w*), the void fraction could be reduced from 0.06 to 0.01 [15]. Interestingly, as previously mentioned, only TL LS saw significant reductions in void fraction with the addition of monostearate, while TL/M RS had similar properties to its pure counterpart even at a lower solid fat content. Likewise, previous trends were not observed when comparing CB samples and their respective SFC values (Table 1), which revealed that CB RS had the lowest SFC (74.3%) yet the highest void fraction (0.114). In the prior work mentioned, TL/triolein mixtures had extreme differences in SFC compared to pure TL with corresponding samples containing 20% and 40% oil differing in SFC by as much as 56% and 78%, respectively. In this work, these kind of deviations in SFC were only observed when TL samples were compared to corresponding CB samples; doing so revealed large differences in SFC (20% or more) in addition to TL networks generally having much higher void fractions than the respective CB samples. This result may suggest that differences in phase ratio will only show proportional effects on the quantity of voids, as previously reported, when large deviations are observed throughout crystallization processes.

Interestingly, comparisons between cocoa butter and trilaurin containing monostearate exhibited different effects of emulsifiers on their void morphology. While 3D images in Figure 4a,b showed similarities between the topology of CB/M LS samples and those produced with TL, the dissimilar morphology observed between their CT image slices at the surfaces and cross-sections of voids may indicate the voids of CB/M LS were not formed under sintering mechanisms. Lencki and Craven (2013) suggested that over tempering CB at higher temperatures can lead to the development of highly porous or low-density fat networks. They attributed the formation of this network to negative pressure zones (or sintering) similar to the case in TL [25]. Likewise, sintering during crystallization typically results in a significantly stronger crystalline network due to sintering bond formation [38]. However, in this study, CB/M LS was not crystallized under tempering conditions and formed a highly brittle structure compared to all other samples. Thus, it is hypothesized that instead of the voids of CB/M LS being formed from sintering mechanisms, the structure encountered some form of internal stress during post-process crystallization. Such stress can lead to a relaxation event due to which crack formation and subsequent propagation occurs [51]. Further investigations would be necessary to confirm this theory and determine the mechanism and attributes that make CB/M LS samples vulnerable to cracking.

## 3. Materials and Methods

### 3.1. Materials

Cocoa butter (CB) was donated by Cargill Cocoa & Chocolate (Cargill, West Chester, PA, USA). Trilaurin (TL) and glyceryl monostearate (M) were purchased from TCI America (Portland, OR, USA) and Combi-Blocks (San Diego, CA, USA), respectively. TL had >98.0% purity whereas M had a purity of ≥95%.

### 3.2. Crystallization Process

Two lipid systems (cocoa butter and trilaurin) were selected as base lipids, and glyceryl monostearate was used as an emulsifier additive. Each oil sample was blended with M at 0 and 5% (*w*/*w*). Monostearate was added at 5% (*w*/*w*) because it has been shown to alter crystallization kinetics between 2% and 8% (*w*/*w*) [29]. Blends were heated to 70 °C and held for at least 30 min to remove crystal memory. Molten oil blends were crystallized using an agitator mixer (Model BDC3030, Caframo Limited, Wiarton, Ontario, Canada) and a temperature-controlled shear cell previously described by Maleky and Marangoni (2011) [6]. To produce eight different crystallized samples, each oil sample was sheared under random (RS) and laminar (LS) shear using a pitched propeller blade and a Couette-type shear cell attachment, respectively. During this process, samples were isothermally crystallized from 60 °C while being sheared at approximately 500 s^−1^ at a cooling rate of 2 °C/min. Prior to the analysis of samples, CB-based samples were incubated at 20 °C for four days, and TL based samples were stored at 30 °C for 24 h. CB samples were incubated for four days to ensure the stability of sample polymorphs in addition to maintaining the consistency of samples for later comparisons. To further elucidate the impact of shear on the porous properties of lipids, an additional set of CB samples was developed under 100 s^−1^. For clarity, in this study, CB and TL samples without monostearate processed under RS or LS at 500 s^−1^ are referred to as CB RS, CB LS, TL RS, and TL LS, respectively. Samples containing 5% monostearate are named CB/M RS, CB/M LS, TL/M RS, and TL/M LS. CB samples crystallized under 100 s^−1^ are labeled similarly except their names are followed by ‘100′, indicating their respective shearing rate.

### 3.3. Solid Fat Content

Using pulsed nuclear magnetic resonance (NMR) (Minispec MQ20 Spectrometer, Bruker Optics Ltd., Milton, ON, Canada), the solid fat content (SFC) of lipid samples were determined by measuring the proportion of signal corresponding to the relaxation of protons in the solid state of samples. Approximately 2–3 mL of each incubated sample was transferred into glass NMR tubes (10 mm diameter, 1 mm thickness, and 180 mm height), and their SFC was measured at a minimum in triplicate.

### 3.4. Thermal Properties

The thermal properties of the crystalized samples were analyzed using a Differential Scanning Calorimeter (DSC) (Q2000, TA Instrument, New Castle, DE, USA). Approximately 5–10 mg of incubated sample was hermetically sealed inside DSC pans. The melting properties of samples were then measured using a heating regime set at 1 °C/min. Cocoa butter and trilaurin samples were heated between 20 and 50 °C, and 30 and 65 °C, respectively. Sample melting points were determined by finding the peak heat flow for each respective sample using TA Universal Analysis 2000 software (TA Instrument, New Castle, DE, USA). Samples were measured at a minimum in triplicate.

### 3.5. Crystal Morphology

Using a Zeiss Axio Imager Upright Microscope (Carl Zeiss Microscopy GmbH, Jena, Germany), polarized light microscope (PLM) images were taken of the crystal microstructure of lipid samples. Samples were prepared by placing a small portion of crystallized lipid on a preheated glass slide at 20 or 30 °C for CB and TL samples, respectively. Lipid samples were then gently compressed using a preheated coverslip and analyzed. Images were converted to 8-bit grayscale images using ImageJ software and segmented using the Li auto threshold command to isolate the crystals in the network and measure their average crystal size (μm^2^). A minimum of four images were taken from three replicate slides for image analysis.

### 3.6. Polymorphic Behavior

The polymorphic behavior of lipid samples was determined using a Rigaku Miniflex 600 Powder Diffractometer (Rigaku, Tokyo, Japan) fitted with an X-ray copper tube set to a voltage and current of 40 kV and 15 mA, respectively. Samples were prepared by placing a small portion of crystallized lipid on top of preheated glass sample holders. Samples were analyzed at 1°min^−1^ (° = 2θ) with measurements being taken at every 0.02° between 5° and 40°. D-spacings associated with the short spacings between crystallographic planes of samples were computed by analyzing diffractogram peaks using PDXL 2.8.4.0 software (Rigaku, Tokyo, Japan). A minimum of two replicates were analyzed per sample.

### 3.7. Pore Network Measurements

**Figure 7:**
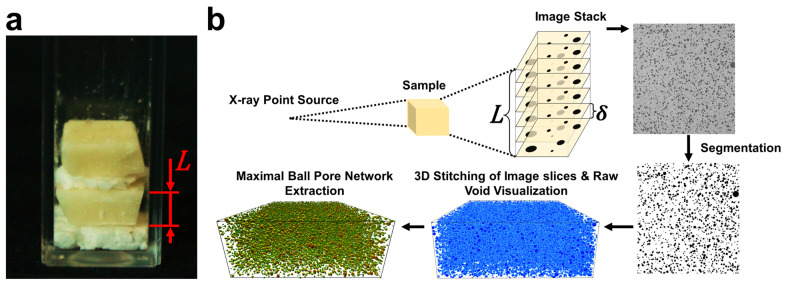
(**a**) A representation of one sample prepared for CT analysis which contains two replicates (with thickness *L*) separated by Styrofoam. (**b**) The CT analysis workflow.

The pore network properties of lipid samples were imaged using X-ray microcomputed tomography (CT) with a Zeiss Xradia 510 Versa 3D X-ray Microscope (Carl Zeiss Microscopy GmbH, Germany). Replicates were prepared for analysis by cutting crystallized samples into approximately 10 × 10 mm^2^ square slabs and carefully stacking up to three replicates inside a plastic cuvette with a length, width, and height of 12.5 mm × 12.5 mm × 45 mm and a thickness of 1.25 mm. To better differentiate replicates, a thin compressed layer of Styrofoam was placed in between each slab (shown in Figure 7a).

To protect CB samples from melting, they were sealed and placed inside a small Styrofoam bath filled with 20 °C tap water when analyzed. The X-ray was set to a voltage of 60 kV and 2401 projections were taken as samples were rotated 360°. From these projections, images along the axial direction were obtained, with image resolutions and the quantity of images representing replicates ranging from 14.66 to 17.69 μm/pixel and from 100 to 150 images, respectively. Images were then segmented using MIPAR image analysis software (MIPAR Software LLC, Worthington, OH, USA). The segmentation was performed using an optimization technique that finds the pixel intensity distribution minimum between the solid/liquid region and the void. We used a combination of thresholding techniques only in the case of TL LS and TL/M LS samples because they had smaller voids that resulted in less distinct pixel intensities between phases.

**Figure 8:**
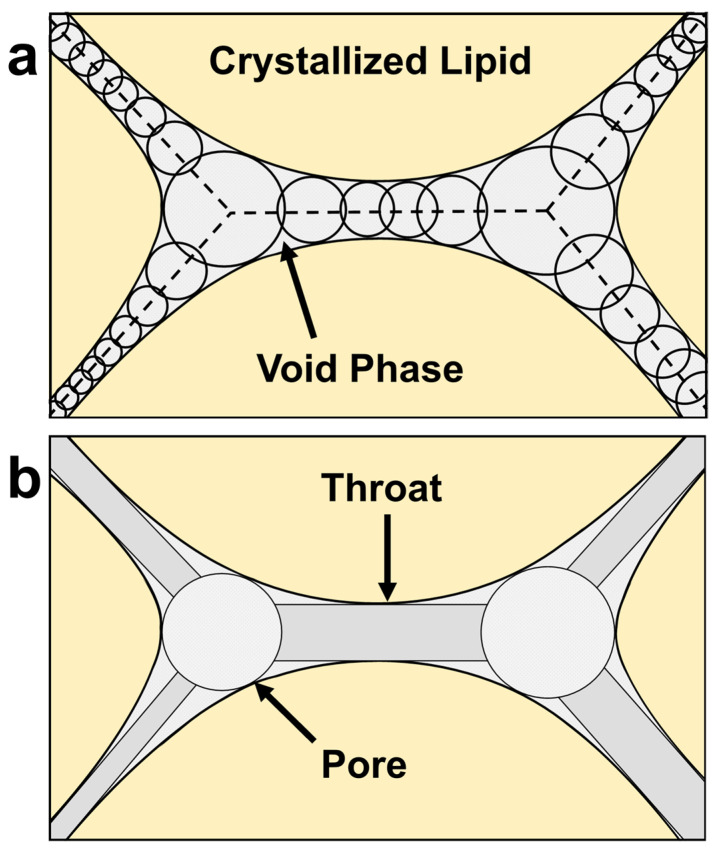
A depiction of how the MB algorithm discretizes the void phase of lipids from (**a**) maximally inscribed spheres along the medial axis (dashed lines) into (**b**) pores and throats.

The maximal ball algorithm (MB), first developed by Silin and Patzek (2006) and sequentially by several others, provided a robust method for the extraction of pore morphological features from high-resolution 3D CT images [41,53,54,55]. The algorithm finds the distance (also called the maximally inscribed sphere radius) between the center of a specified void voxel and the nearest solid boundary. Using this information, the algorithm removes any maximal balls that are redundant. This leaves behind a discretized pore space made up of maximally inscribed spheres along the medial axis of the void as shown in Figure 8a. By knowing the maximally inscribed sphere radius for all void voxels along the medial axis, hierarchal relationships between neighboring voxels and their maximally inscribed radii are made. Using these relationships, the morphology of the void can be described discretely using pores (spheres) and throats (cylinders) for a wide range of topologies (Figure 8b). In this study, the MB algorithm was utilized for the analysis of CT images using the open-source MB algorithm package made available by Raeini (2019) [56]. The pore and throat network extractions were performed on segmented images via an executable, pnextract. The data obtained from the extraction were sorted and analyzed using an algorithm developed in the python programming language (Python Software Foundation, https://www.python.org/, accessed on 10 December 2020). Moreover, using the VPython 7 programming package, a 3D visualization of pore and throat data was created [57]. An example of the sample analysis workflow using CT all the way through the image analysis can be found in Figure 7b. A minimum of three replicates were taken per sample except for TL/M RS samples which only had two.

### 3.8. Statistical Analysis

Statistical analysis was performed by comparing the means of samples using ANOVA. For categories of data satisfying ANOVA assumptions, ordinary two-way ANOVA was employed using Tukey’s multiple comparison test. For heteroscedastic data Brown–Forsythe and Welch ANOVA tests were employed using the Dunnet T3 multiple comparisons test. All data considered statistically significant obtained an α ≤ 0.05. Statistical analysis was done using GraphPad Prism 8.4.1 software (GraphPad Software, Inc., San Diego, CA, USA). For averages reported in tables made of distribution data such as crystal size, connectivity, and pore and throat radius, the average was obtained by finding the average value of each replicate distribution and then taking the average of all replicates.

## 4. Conclusions

Overall, the results found in this study demonstrate that, as predicted, the morphology and quantity of the voids in crystallized lipids is greatly impacted by both the applied shear and the composition. Typically, the higher the void fraction of a material, the higher the connectivity that can be observed [47,55]. However, this phenomenon was not consistent across all samples produced here. This inconsistency was indicated by the low connectivity yet higher void fraction of CB RS samples. On the other hand, CB/M LS cocoa butter samples had a much lower void fraction and the highest connectivity, likely due to crack formation. TL samples, in contrast to CB, had generally higher void fractions and connectivity that were moderately correlated. Due to the similar pore properties observed across all TL samples, the linear increase in connectivity with corresponding increases in void fraction suggests that pore networks developed by sintering mechanisms within lipids form a positive relationship. This relationship was further confirmed by CB networks that clearly had several mechanisms driving the formation of the voids and were not correlated even when regression was performed on samples with similar morphology (CB RS, CB LS, and CB/M RS). In terms of pore sizes, these findings showed that corresponding pore sizes of lipid samples developed from the same base fat were primarily affected by the type of shear and shearing rate applied rather than by the composition. This result corroborates previous findings that have shown air/oil foams crystallized in the presence of emulsifier under high shearing rates to develop networks with similar pore sizes independent of emulsifier concentration [30].

## Figures and Tables

**Table 1:** Crystal size and solid fat content (SFC) measurements for cocoa butter (CB) and trilaurin (TL) samples.

Sample	Crystal Size (μm^2^) *	SFC (%) *
CB RS	8.0 ^A^ ± 0.5	74.3 ^A^ ± 1.0
CB LS	8.5 ^A^ ± 0.4	76.4 ^B, C^ ± 0.4
CB/M RS	13.3 ^B^ ± 2.6	75.9 ^C^ ± 0.9
CB/M LS	11.4 ^B^ ± 0.8	77.4 ^B^ ± 0.6
TL RS	18.3 ^a^ ± 1.4	98.2 ^a^ ± 0.1
TL LS	13.9 ^a^ ± 4.2	97.8 ^b^ ± 0.0
TL/M RS	13.6 ^a^ ± 1.1	94.1 ^c^ ± 0.6
TL/M LS	18.6 ^a^ ± 5.1	95.4 ^c^ ± 0.1

* Mean values for CB and TL followed by different uppercase and lowercase superscript symbols, respectively, within the same column indicates statistical significance (α ≤ 0.05) between samples made of the same base fat (CB or TL). Errors reported were computed from the sample standard deviation.

**Table 2:** Pore connectivity (z), volume weighted pore and throat radii (R_43_), and void fraction (v).

Sample	Connectivity *	Pore Radius *	Throat Radius *	Void Fraction *
	z	R_43,p_ (μm)	R_43,t_ (μm)	v
CB RS	0.39 ^A, B^ ± 0.20	50.8 ^A^ ± 2.8	34.4 ^A^ ± 3.2	0.114 ^A^ ± 0.029
CB LS	0.42 ^A^ ± 0.12	50.0 ^A^ ± 3.9	44.4 ^A^ ± 6.8	0.019 ^B^ ± 0.018
CB/M RS	0.10 ^B^ ± 0.05	40.2 ^A^ ± 8.0	32.4 ^A^ ± 4.5	0.022 ^B^ ± 0.011
CB/M LS	1.88 ^C^ ± 0.37	45.4 ^A^ ± 17.7	28.8 ^A^ ± 10.6	0.046 ^B^ ± 0.027
TL RS	1.87 ^a, b^± 0.12	61.2 ^a^ ± 3.5	33.81 ^a^ ± 5.8	0.118 ^a, b^± 0.012
TL LS	2.44 ^a^ ± 0.62	31.8 ^b^ ± 6.8	18.01 ^b^ ± 3.6	0.139 ^a^ ± 0.034
TL/M RS	1.92 ^a, b^ ± 0.10	57.3 ^a^ ± 2.9	36.02 ^a^ ± 1.8	0.120 ^a, b^ ± 0.006
TL/M LS	1.05 ^b^ ± 0.38	24.2 ^b^ ± 4.2	12.32 ^b^ ± 3.1	0.087 ^b^ ± 0.014

* Mean values for CB and TL followed by different uppercase and lowercase superscript symbols, respectively, within the same column indicates statistical significance (α ≤ 0.05) between samples made of the same base fat (CB or TL). Errors reported were computed from the sample standard deviation.

## Data Availability

Data can be made available upon request.

## Sample Availability

Samples used for analysis in this study are not available from the authors.
